# The brains of aged mice are characterized by altered tissue diffusion properties and cerebral microbleeds

**DOI:** 10.1186/s12967-020-02441-6

**Published:** 2020-07-08

**Authors:** Erik N. Taylor, Nasi Huang, Jonathan Wisco, Yandan Wang, Kathleen G. Morgan, James A. Hamilton

**Affiliations:** 1 Department of Radiology, University of New Mexico, Albuquerque, NM USA; 2 Department of Physiology & Biophysics, Boston University School of Medicine, Boston, MA USA; 3 Department of Biomedical Engineering, Boston University, Boston, MA USA; 4 Department of Anatomy & Neurobiology, Boston University School of Medicine, Boston, MA USA; 5 Department of Health Sciences, Boston University, Boston, MA USA

**Keywords:** Brain imaging, Aging, Cerebral microbleeds (CMBs), Vascular contributions to cognitive impairment and dementia (VCID), Gradient-recalled echo MRI, diffusion tensor imaging

## Abstract

**Background:**

Brain aging is a major risk factor in the progression of cognitive diseases including Alzheimer’s disease (AD) and vascular dementia. We investigated a mouse model of brain aging up to 24 months old (mo).

**Methods:**

A high field (11.7T) MRI protocol was developed to characterize specific features of brain aging including the presence of cerebral microbleeds (CMBs), morphology of grey and white matter, and tissue diffusion properties. Mice were selected from age categories of either young (3 mo), middle-aged (18 mo), or old (24 mo) and fed normal chow over the duration of the study. Mice were imaged in vivo with multimodal MRI, including conventional T2-weighted (T2W) and T2*-weighted (T2*W) imaging, followed by ex vivo diffusion-weighted imaging (DWI) and T2*W MR-microscopy to enhance the detection of microstructural features.

**Results:**

Structural changes observed in the mouse brain with aging included reduced cortical grey matter volume and enlargement of the brain ventricles. A remarkable age-related change in the brains was the development of CMBs found starting at 18 mo and increasing in total volume at 24 mo, primarily in the thalamus. CMBs presence was confirmed with high resolution ex vivo MRI and histology. DWI detected further brain tissue changes in the aged mice including reduced fractional anisotropy, increased radial diffusion, increased mean diffusion, and changes in the white matter fibers visualized by color-coded tractography, including around a large cortical CMB.

**Conclusions:**

The mouse is a valuable model of age-related vascular contributions to cognitive impairment and dementia (VCID). In composite, these methods and results reveal brain aging in older mice as a multifactorial process including CMBs and tissue diffusion alterations that can be well characterized by high field MRI.

## Background

The growing geriatric population (49.2 million individuals in the USA in 2016 [1]) poses a significant social, economic, and health care burden with growing costs [2]. As life expectancy increases in developed countries, the development of the major debilitating and life-threatening conditions with age as the primary major risk factor increase in prevalence, including cardiovascular disease and neurodegeneration [3]. Of intense concern is the aging brain, with Alzheimer’s disease (AD) and related dementias, including vascular dementia, becoming ever more common and requiring substantial resources to prevent, diagnose, treat, and manage [4]. Small vessel disease, a particular cause of subcortical vascular dementia affecting the small cerebral blood vessels, may account for an estimated ~ 50% of all dementias worldwide [5].

Since AD or vascular dementia is currently untreatable, research has focused on differentiating healthy brain aging from early disease processes. Magnetic Resonance Imaging (MRI) is a clinically applicable tool that can be used to gain insights into brain aging or neurological disease progression [6]. In particular, structural MRI is established as a baseline measure of brain integrity and may eliminate other etiologies in combination with neurological presentation. During aging, regions of the brain involving memory and cognition shrink, with age related differences being largest for total brain, frontal lobe, and medial temporal brain volumes [7–9] followed by further neocortical neuronal loss occurring with late-stage disease [10]. Multimodal MRI, the coordinated use of multiple mutually informative probes to understand brain structure and function, [9, 11, 12] including diffusion-weighted imaging (DWI) and gradient-recalled echo (GRE) MRI with T2*W, offers additional insights into the progression and mechanisms of brain aging.

In neural tissue, water diffusion is restricted by axonal bundles and myelin sheaths, resulting in diffusion patterns that are predictable along the direction of white matter fiber tracts [13–15]. During the aging process, the breakdown of ordered myelinated neural structures occurs, resulting in less restricted water diffusion in DWI. Diffusion tensor imaging (DTI) is a related acquisition and analysis MRI technique that can measure white matter disease by detecting increasing anisotropy and unrestricted water diffusion with AD disease progression [16]. Although another structural technique, Fluid Attenuated Inversion Recovery (FLAIR), exhibits the presence of edematous events in white matter, DTI is quantitative, offering improved pathological specificity for assessing early degenerative stages [6, 9].

AD and dementia progression is a multifactorial process and can progress with a cerebrovascular phenotype. Such vascular changes are visible with MRI. For example, CMBs, also known as microhemorrhages, are biomarkers of aging, hypo-intense on GRE MRI, associated with geriatrics, cognitive impairment, and risk of stroke [17–20]. As the stiffness of the proximal aorta increases with age, [21–23] high pulses of pressure are sent to the small delicate blood vessels downstream. These pulses could damage the cerebral vessel walls in a way that increases leaking of blood into vulnerable brain regions, a possible mechanism for CMBs formation [24, 25]. Depending on the location, CMBs may indicate neurovascular or neurologic disease. Lobar CMBs are associated with cerebral amyloid angiopathy, [26] while deep or mixed CMBs are associated with hypertensive arteriopathy [20, 27, 28]. While CMBs are chronic biomarkers of neurovascular disruption, their direct role in AD is still a matter of debate. A recent study by Nation et al. found that acute blood–brain barrier (BBB) breakdown in MRI is an early predictor of human cognitive dysfunction that may be a unique component of AD progression independent of p-Tau or Aβ [29].

The mouse is an established model appropriate for investigating brain aging using MRI [30, 31]. A comparison of age-related changes in cognition in laboratory animals can help disambiguate the boundary between normal and pathological states of aging in humans [32] with aged rodents being commonly used in cognitive research [33]. As in humans, grey matter rich regions decline with age, white matter changes occur, and increases in ventricle cerebrospinal fluid (CSF) are observed [31]. Laboratory animals are used routinely because age related cognitive decline and behavioral alterations mimic similar pathophysiology of human AD, albeit at a more rapid time scale, particularly with the use of genetically modified animals [34]. However, (1) there is a lack of rodent studies aged-matched to human counterparts [30], and (2) the presence of CMBs in vivo, in the context of age-matched vascular aging, has not been shown in mice. For these reasons, identification of a rodent model of CMBs formation in normal aging is novel and representative of human cerebrovascular disease.

The objective of this study was to use a murine model to relate structural changes that occur in the brain during aging with other features in multimodal MRI, including tissue microstructural properties and the presence of CMBs in older mice. These multimodal MRI methods supplement standard T1W or T2W MRI measurements with alternate approaches that provide high specificity for the diffusion microarchitectural features of the brain and iron content, using DTI and T2*W, respectively. Following the identification of CMBs in vivo, ex vivo T2*W high resolution MR-microscopy was used with subsequent histopathology characterization. DTI was collected ex vivo and then used to calculate diffusion tensors that were either analyzed directly or modeled across the entire brain with tractography. The small size of the mouse brain and the availability of post-mortem tissues offers optimal conditions to characterize brain microstructural features and CMBs with ex vivo imaging, while also providing a valuable model of age-related neurovascular biomarkers for future routine investigations.

## Methods

### In vivo MRI

Mice were imaged with a multimodal MRI protocol on a Bruker 500 MHz 11.7T system (MRI; Bruker Co., Billerica, MA) at the Boston University Medical Campus MRI/NMR High Field Imaging Core. We acquired T2W (RARE; TR = 2500 ms, TE = 26 ms, flip angle = 180°, 0.1 × 0.1 mm^2^ in plane resolution with 1 mm slice thickness) and T2*W (FISP; TR = 1528.02, ms TE = 4 ms, excitation pulse angle = 15°, 140 µm^3^ isotropic voxels) images in vivo. Three age groups of male C57BL/6J (Jackson Laboratory, Farmington, CT, USA) mice were used for the in vivo study: 3 mo, 17–18 mo, and 24–25 mo. These groups were defined as young, middle-aged, and old groups [30] with 4 young, 3 middle-aged, and 3 old mice used for MRI. The mice were anesthetized with 0.5–2% isoflurane gas under oxygen flow and stabilized in the central area of the magnetic field with a 30 cm RF volume coil devoted to mouse imaging.

### Ex vivo MRI

After completion of the in vivo imaging protocol, the mice were sacrificed under anesthesia and perfused transcardially with 0.01 M phosphate buffered saline (PBS) through the left ventricle for 3 min at a rate of 2 ml/min (Rainin peristaltic pump) followed by 4% paraformaldehyde (PFA) solution. The brain was then isolated from each mouse. High resolution imaging was repeated overnight in a 15 cm RF volume coil with freshly excised brains. For ex vivo DTI, a total of 91 diffusion sampling directions were acquired (3D-EPI; TR = 750 ms, TE = 19.6 ms, number of segments = 5, diffusion duration = 2.4 ms, diffusion separation = 9 ms, 175 µm^3^ isotropic voxels) at a constant b-value of 2000 s/mm^2^.

Additional old-aged (21–24 mo) female C57BL/6 J mice were included for high resolution ex vivo imaging emphasizing the T2*W protocol for detection of CMBs with brains placed in MRI signal inert fomblin Y (Sigma-Aldrich, Saint Louis, MO). For these additional ex vivo only mice, brains were soaked in 1% (v/v) Magnevist in saline for 5 days prior to ex vivo imaging [35]. High resolution MR-microscopy with T2*W was performed using the same in vivo GRE sequence of 3D fast imaging with steady-state free precession (FISP) only at higher resolution ex vivo (55 µm^3^). A particularly important feature of GRE imaging is that gradient reversal only affects those spins that have been dephased by the action of the gradient itself, so magnetic field inhomogeneities, including those from CMBs, are not cancelled. For validation of CMBs presence, N = 16 brains total were included in the ex vivo T2*W study.

### Image analysis

Cortical thickness was measured superior to the corpus callosum (Cc) and the hippocampus (Hi) within coronal in vivo T2W MR images, starting at the most caudal location where the Cc was visible, with 12 linear measurements in multiple locations and across multiple slices per animal. Ventricle area was calculated by generating a maximum intensity projection (MaxIP) from slices that contained the lateral ventricles (5 to 6 slices) with in vivo T2W MRI. Use of the MaxIP prevented the overestimation of ventricle size due to partial volume effects. Quantification was carried out by drawing a region of interest (ROI) in the bright areas of the two lateral ventricles on the projection image resulting in readings from the left and right sides for each mouse. Quantification of these brain structures was carried out in ImageJ software (NIH, Bethesda, MD).

CMBs were manually counted in terms of the volume and number. Hemosiderin in blood deposits from chronic brain microhemorrhages (another name for CMBs) and is a strong paramagnetic material that can be imaged in GRE MRI with T2*W. CMBs appeared as hypo-intense round or ovoid objects in the T2*W images in specific anatomic brain regions, as described previously [36]. Images were displayed as either single slices for quantification and comparison with histology, or as projections using a minimum-intensity projection (MinIP) for visualization of multiple slices. Counting was performed using ImageJ software in individual slices within the principle imaging planes to confirm the CMBs 3D shape and to rule out misidentification of normal blood vessels that are linear and present across multiple slices.

Advanced Normalization Tools (ANTs) software was used on ex vivo T2*W images for voxel based morphometry, a whole-brain technique for characterizing regional brain volume differences and differences in tissue concentration, particularly grey matter, across subjects [37]. Image analysis was performed with pre-processing (*N3BiasFieldCorrection*) and linear co-registration (*antsRegistration*), followed by non-linear registration (*antsRegistrationSyN*), and study template generation (*buildtemplateparallel*). Segmentation of grey matter and white matter (*Atropos*) and calculation of Jacobian determinants (*ANTSJacobian*) was performed on individual brain images. Statistical analysis was carried out in FMRIB Software Library (FSL) on concatenated 4D datasets (*fslmerge*) followed by an unpaired *T* test comparison (*randomize*).

Diffusion tensor imaging (DTI) processing methods were used to produce voxel-based measurements of fractional anisotropy (FA), mean diffusion (MD), and radial diffusion (RD) using ex vivo brains. ROI placed in multiple slices of the thalamus and parietal–temporal cortex were used to quantify DTI metrics. Generalized Q-space imaging (GQI), a model-free diffusion data processing method, was used for data visualization and tractography generation with DSI studio software (Fang-Cheng Yeh; University of Pittsburgh) with the color of tracts encoded to local DTI measurement values [38].

### Histology

Following ex vivo MRI, brains were post-fixed with 4% PFA over two nights, then placed in 15% and then 30% sucrose. The brains were frozen in isopentane with dry ice and cryo-sectioned with 50 µm thickness at the coronal plane. Brain tissues were then stained with a mixture of 4% potassium ferrocyanide and 4% hydrochloric acid (Iron Stain Kit, Millipore-Sigma, Burlington, MA) for Prussian Blue staining and then co-stained with Nuclear Fast Red for tissue structure and background.

### Ex vivo MRI and microCT vascular atlas

The brain MRI and microCT vascular atlas was analyzed from ex vivo data acquired by Dorr et al. [39] in male CBA mice. For MRI, a 7.0-T MRI scanner (Varian Inc., Palo Alto, CA, USA) was used with skulls placed into proton-free susceptibility-matching fluid (Fluorinert FC-77, 3M Corp., St. Paul, MN, USA). The parameters used in the scans were optimized for grey/white matter contrast: T2W, 3D fast spin-echo sequence, with TR/TE = 325/32 ms, four averages, field-of-view 12 × 12 × 25 mm^3^ and matrix size = 780 × 432 × 432 resulting in an image with 32 μm isotropic voxels. For microCT (GE Healthcare, Chicago, IL, USA) the brains were removed from the skulls and mounted in 1% agar. Each vascular image volume was acquired with 20 μm isotropic resolution using the GE eXplore Locus SP specimen scanner. Images were obtained from 720 views over a 360° rotation in 2 h with an X-ray tube current of 80 μA and voltage of 80 kVp.

### Statistical methods

Statistical analyses were carried out with the T-test for pairwise comparison and with other statistical models. For statistical analysis involving multiple groups, RStudio was used to perform a one-way ANOVA test, where a significant p-value indicated that some of the group means are different. This was followed by Tukey Honest Significant Differences in R for performing multiple pairwise-comparison between the means of groups.

## Results

### In vivo structural MRI

**Fig. 1:**
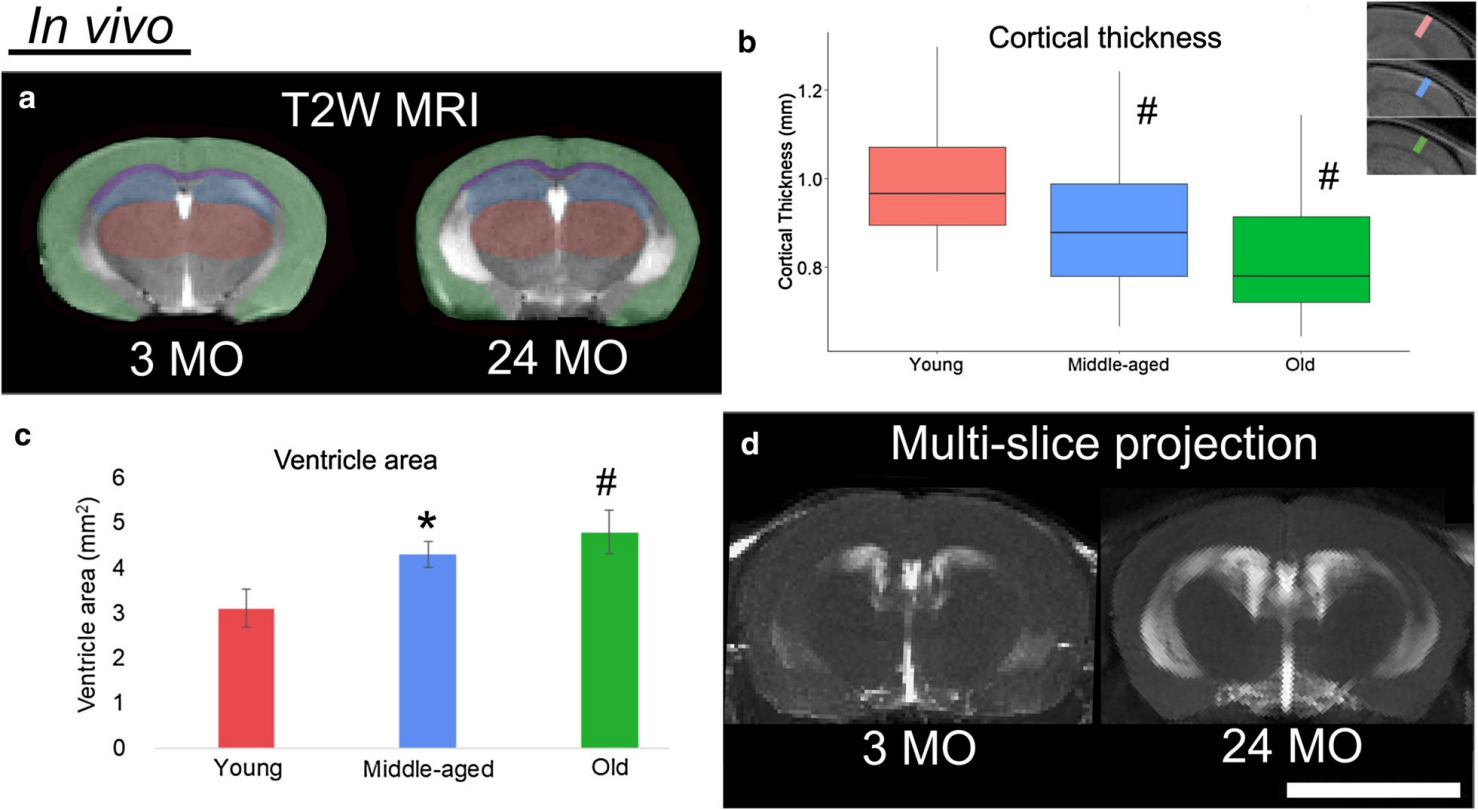
Changes in brain structure occurring with aging in C57BL/6J mice. In vivo T2W MRI images are shown with single-slices from young (3 mo) or old (24 mo) mice in **a** and multi-slice maximum intensity projections (MaxIP) in **d**. The single-slice rostral view (**a**) is labeled with the cortex (Ct; green), hippocampus (Hi; blue), thalamus (red) and corpus callosum (Cc; purple) visible from a young and old mouse. **b** Quantification was carried out as linear measurements of Ct thickness superior to the Hi and Cc, across multiple locations and slices. Examples of Ct thickness measurements in the respective age groups are shown (top young mouse; bottom old mouse) with inset left hemisphere region, where the young mouse Ct thickness was about 1 mm. **c** The area of the two lateral ventricles was measured from the MaxIP images (*p value is less than 0.05 and ^#^p value is less than 0.01 by ANOVA followed by Tukey multiple pairwise-comparisons). The white bar in **d** represents a scale bar with size of 5 mm

The aging brains of the mice were imaged using a multimodal MRI protocol including various acquisition protocols and image weightings. Brain aging was visible with MRI in conventional T2W images in terms of brain structure and CSF presence in the lateral ventricles. Compared to the young mice, the brains of middle-aged and old mice demonstrated grey matter loss, as measured by cortical thinning superior to the Hi and Cc with aging (Fig. 1). Enlargement of the ventricles was clearly observed in multi-slice MaxIP images, a further indication of reduced brain-tissue volume in middle-aged or old mice.

### Detection of CMBs in vivo

**Fig. 2:**
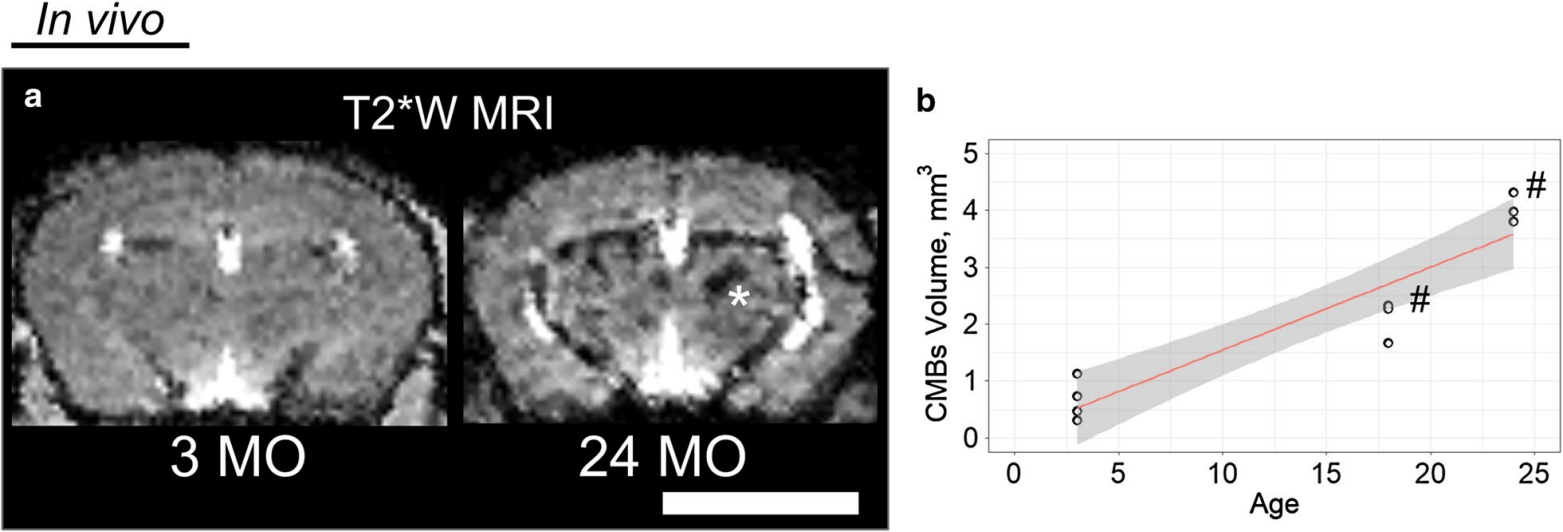
T2*W MRI located the hemosiderin deposits of CMBs in vivo. **a** A large CMB region was visible in the old-aged mouse is labeled with * in the MRI slice shown. **b** The relationship found between CMBs presence visible in T2*W MRI and the age of mice in months was significant in the linear regression model (p < 0.001; R^2^ value of 0.8716). The volume of CMBs was measured in vivo manually as the area of CMBs on an image multiplied by the slice thickness. Resolution (140 µm^3^ isotropic) was minimized to prevent partial volume effects. Quantification indicated increasing presence of CMBs in relation with aging (# indicates a p value is less than 0.01 by ANOVA followed by Tukey multiple pairwise-comparisons). The white bar represents a scale bar with size of 5 mm

A 3D T2*W acquisition was developed (Methods) for further in vivo analysis. T2*W MRI identified CMBs in the middle-aged and old mice with in vivo images (140 µm^3^ isotropic). CMBs were abundant in middle-age mice and continued to increase in total volume into old-age (Fig. 2).

### Voxel-based morphometry and grey matter analysis ex vivo

**Fig. 3:**
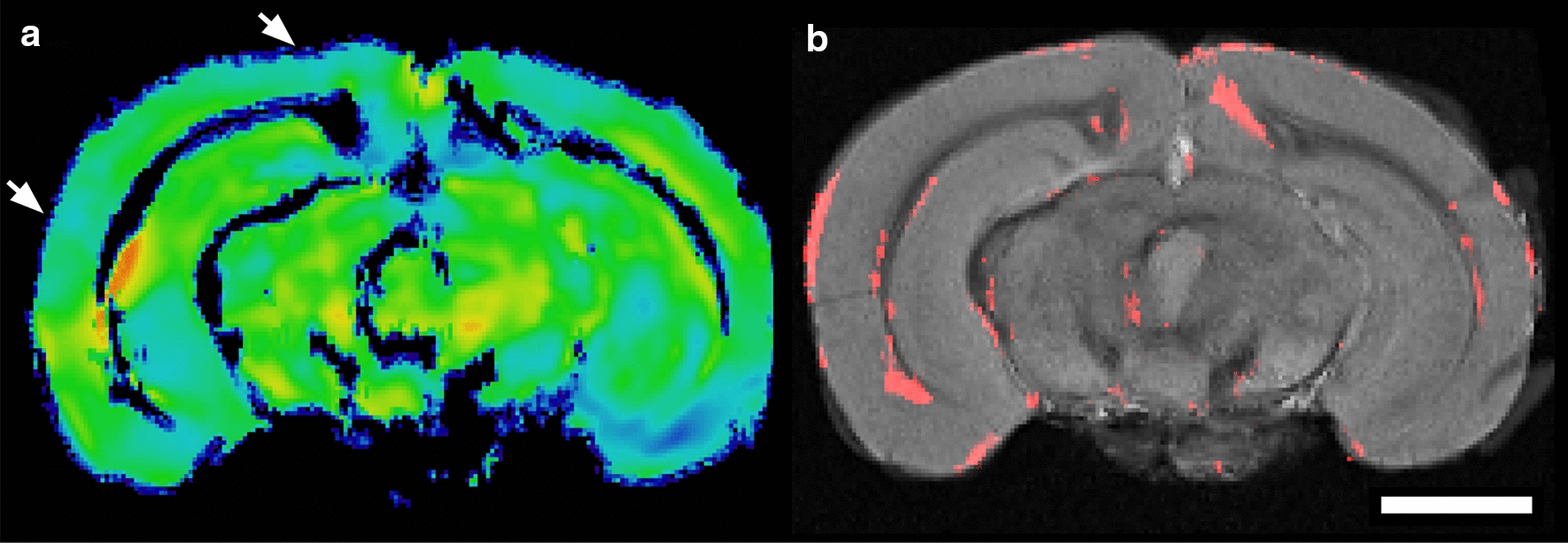
Voxel-based morphometry (VBM) and regional brain analysis. VBM was performed using high resolution ex vivo T2*W MRI, demonstrating a statistical reduction in grey matter volume, particularly in the cerebral cortex. **a** The combined grey matter mask and Jacobian deformation field for an individual old mouse used for VBM analysis after linear and non-linear co-registration and spatial normalization in ANTs. Intensity is mapped to deformation, where red and yellow (positive) or green and blue (negative), respectively, progressively indicated morphologic changes in a representative old-aged mouse. White arrows are representative areas of cortical thinning. **b** A grey-scale template is shown, created from multiple co-registered, normalized, and intensity averaged young mouse brains. The areas where the voxels are significantly different in the young from the old mouse brains, as a combination of the Jacobian Field and grey-matter mask, are overlaid in red, with p < 0.05 (t-test). The white bar represents a scale bar with size of 2 mm

Regional voxel-based morphometry analysis was used to visualize anatomical and volumetric changes in the grey matter of the mouse brain ex vivo. This analysis confirmed the reduction seen in Fig. 1 in vivo in grey matter volume when comparing old aged mice to young mice (Fig. 3).

### Diffusion Tensor Imaging (DTI) of the brain and white matter fiber tractography ex vivo

**Fig. 4:**
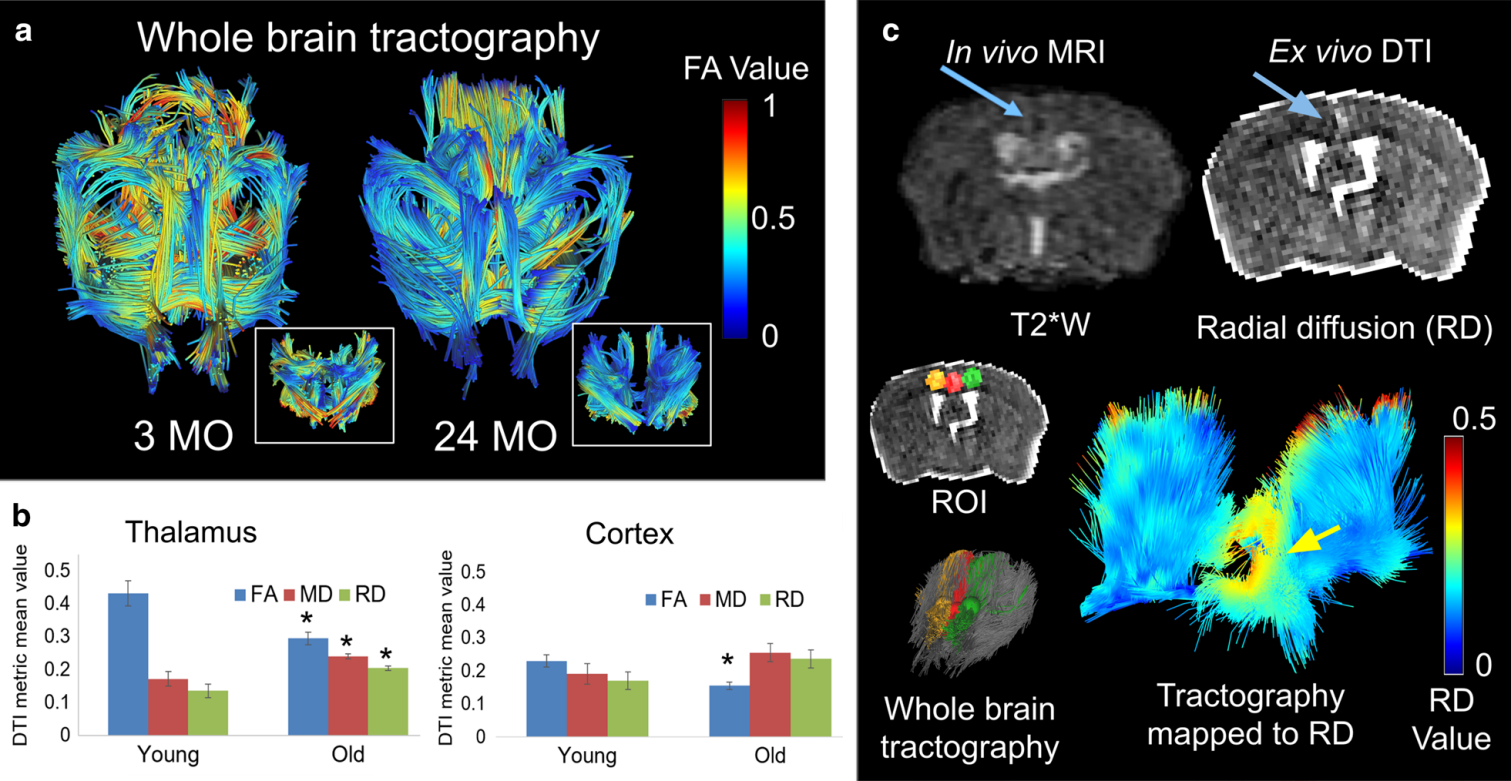
White matter (WM) fiber tractography and DTI changes ex vivo in young versus old C57BL/6J mouse brains. **a** WM fiber tractography in representative 3 mo (Young) and 24 mo (Old) male mice from high resolution ex vivo diffusion MRI scans with B = 2000 s/mm^2^, voxel size of 175 μm^3^, and 91 gradient directions. Whole brain DTI with tractography is shown where FA value is mapped to location. Tractography in the thalamus is displayed in the insets. **b** Measurements of the diffusion properties in the thalamus and parietal–temporal cortex ex vivo. DTI measurements taken from the respective ROI’s included FA, MD, and RD (in units of 10^−3^ mm^2^/s). The FA values in the thalamus/cortex decreased by 68%/67%, MD increased by 140%/134%, and RD increased by 151/139%, respectively, when comparing the 3 mo young mouse brains to old-aged mice (*p value is less than 0.05 when comparing the young mice to the old aged mice group). **c** Anomalous diffusion in diffusion MRI in relation to a CMB site observed with T2*W imaging. The CMB location is marked with an arrow. Radial diffusion (RD) was elevated in brain tissue surrounding the CMB. Local tractography was performed using three regions of interest (ROI) either on the same side (green), contralateral (orange), or through the site of the CMB (red). A region of elevated RD around the CMB region (indicated by the arrow) was found by mapping tractography to RD value (zero is blue and 0.5*10^−3^ mm^2^/s red, scale shown as inset). The white matter tracts passing through the CMBs site were lost. Whole brain tractography in relation to tractography through the ROI sites is shown in inset

Quantitative diffusion measurements were made in the thalamus and cortex ex vivo (Fig. 4). DTI measurements taken in the thalamus and parietal–temporal cortex ex vivo through the placement of ROI included fraction anisotropy (FA), mean diffusivity (MD), and radial diffusivity (RD) values (Fig. 4b). The FA values were decreased in the thalamus, whereas the MD and RD values were elevated in the aged mice cohort compared to the young mice. A hypointense cortical CMB region on T2*W MRI was coincident with abnormal readings in diffusion MRI (Fig. 4c). Regions of interest (ROI) were placed at the site of the CMB, on the same side, or contralateral as shown in the inset of Fig. 4c. Diffusion readings included 30% lower FA and 15% higher MD or 25% higher RD values at the ROI on the CMB side (green) compared to the contralateral side (orange). Tractography at the ROI sites was then performed and was mapped to RD values in order to detect deficits in the white matter (WM) tracts running through the CMB site. A region surrounding the CMB was identified by this method with elevated radial diffusion values and tracts running through the CMB site were deflected and lost.

### CMBs quantification ex vivo

**Fig. 5:**
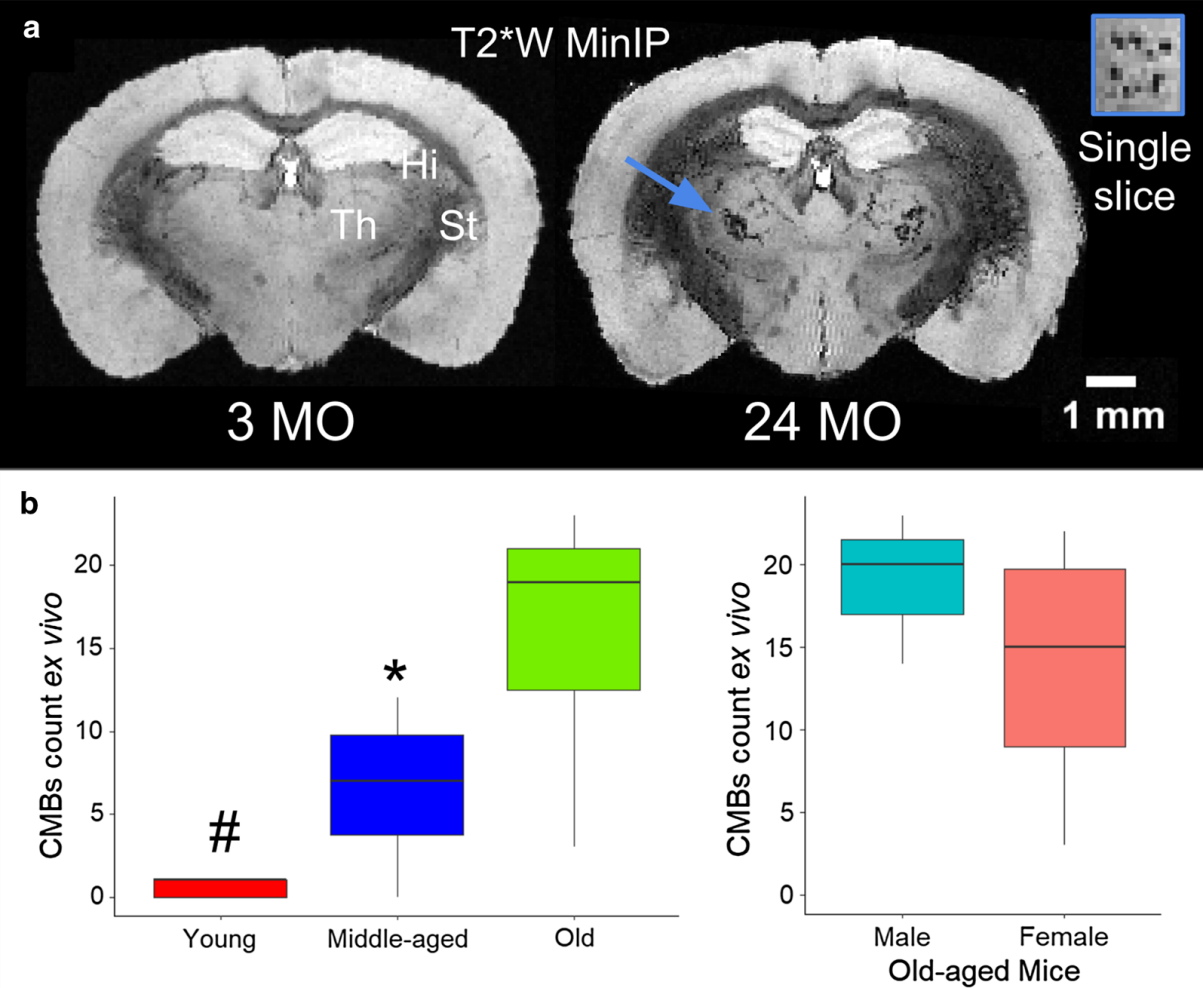
CMBs shown with high resolution T2*W MR-microscopy from ex vivo brains (n = 16 total). **a** The majority of CMBs were found in the thalamus, the relay center of the brain, as shown in a representative minimum intensity projections (MinIP) from multiple slices of T2* MRI ex vivo. The C57BL/6J mice are shown in MinIP with the thalamus (Th), striatum (St), Hi visible from a young (3 mo) and old (24 mo) mouse scanned ex vivo after soaking in Gd contrast for 5 days. The arrow points to multiple thalamic CMBs across multiple slices. A single slice through the region indicated by the arrow is shown as an inset. **b** Multiple-planar views and high resolution isotropic images (55 µm^3^) enabled distinction and counting of individual CMBs. CMBs numbers increased in middle-aged and old mice with no significant differences between the number of CMBs in old-aged mice by sex. ANOVA testing indicated a p value less then 0.01 for CMBs by age, followed by the Tukey multiple pairwise-comparisons test, where ^#^p value of less than 0.01 comparing young-aged to old and *p value of less then 0.05 when comparing middle-aged to old-aged mice for the CMBs count ex vivo

Manual counting of CMBs in high resolution ex vivo T2*W images was carried out across the age groups of C57BL/6 J mice (Fig. 5). Counting of individual CMBs was possible with high resolution ex vivo MR-microscopy using multiple-planar views, avoiding partial volume effects or the misidentification of incompletely perfused blood-vessels. MinIP images showed clusters of CMBs occurring primarily in the thalamus (Fig. 5a). A linear increase in CMBs with age was determined to be a component of aging in middle-aged and old mice of both sexes.

### Histology of CMBs and thalamic blood supply

**Fig. 6:**
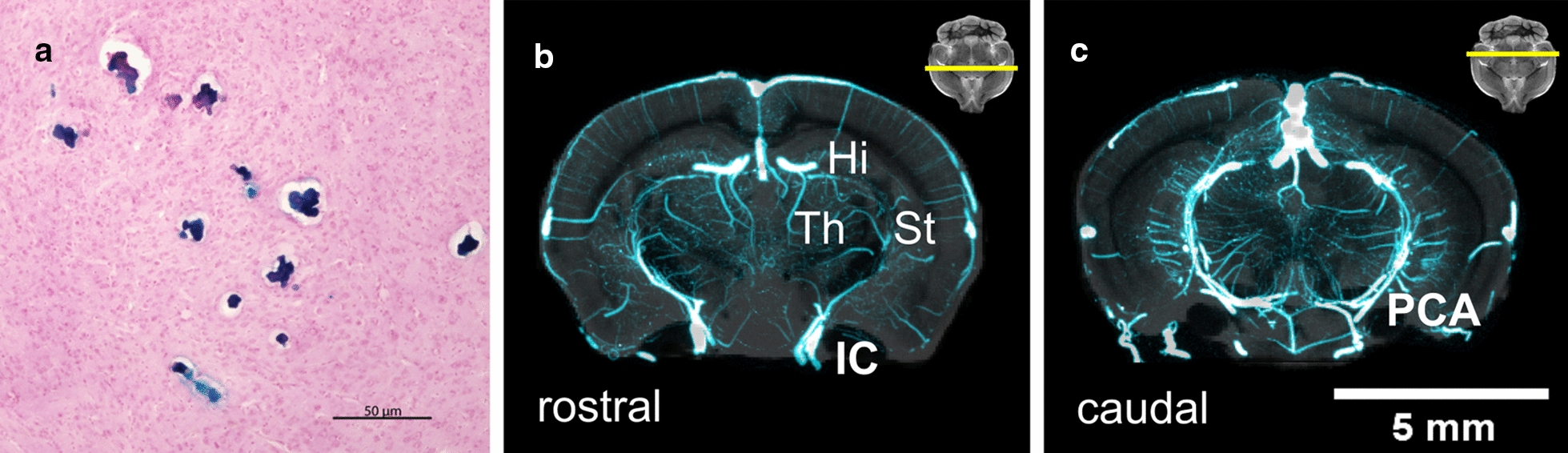
Histologic location of CMBs and analysis of regional blood supply. **a** Prussian blue histology of CMBs in an old mouse with the Th location of CMBs shown. The blood supply in microCT (blue) to the Th shown with MRI (grey) includes **b** the rostral Th, with supply from the internal carotid (IC) with the dorsal and ventral thalamic arteries. The MRI slice location is similar to the location of common Th CMBs, with the Th, St, and Hi labeled from MRI. **c** The blood supply caudal to the Th is shown with the posterior cerebral artery (PCA), connecting via the thalamoperforating arteries. The large central vessels visible superior to the Th in **b**, **c** are longitudinal hippocampal veins. Images in **b**, **c** were reproduced from data acquired by Dorr et al. [39]) using 7.0-T MRI with 32 μm isotropic voxels and microCT with 20 μm isotropic resolution in male CBA mice ex vivo with a scale bar size of 5 mm and the slice location shown as yellow bar on inset horizontal slice

The location of CMBs and local blood supply were examined using histology, microCT, and MRI (Fig. 6). Histology with prussian blue staining was used to identify the CMBs that were in the thalamus of old mice. Vascular anatomy of the thalamus was determined with analysis of an ex vivo atlas containing high-resolution vascular microCT anatomy data co-registered to an MRI brain atlas [39]. The majority of CMBs (as shown in Fig. 5) were located in the atlas region labeled as rostral thalamus (Fig. 6b), with respect to MRI location and blood supply.

## Discussion

### Structural features of brain aging

Many prior studies in mice have focused on transgenic (TG; genetically modified) models of aging with AD disease [31, 34] that is representative of early onset disease [40, 41] and not normal or vascular aging. In these studies, wild-type (WT; non-transgenic) mice were included as side-by-side controls, and so multiple-age comparisons of WT mice were lacking. Longitudinal studies often include younger mice (3–7 mo) [31, 42, 43] and did not include middle-aged or old mice (18–24 mo) that are representative of older age-matched humans [30]. In the longitudinal study by Maheswaran et al. which included transgenic and WT mice, it was found that the WT mouse brain enlarged from 6 to 14 mo, including the whole brain volume, ventricles, and white matter, whereas grey-matter rich regions declined [31]. Of note, studies of aging in mice is well justified because of the access to tissues, the potential for transgenic comparisons, and the lower cost, compared to human studies that require huge study inclusion numbers and an entire life-span to rule out confounding factors [44].

The objective of this study was to determine features of vascular brain aging in middle-aged and old C57BL/6J mice, a common WT strain used for brain research studies, and to compare and optimize MRI methods with results indicative of brain age. Structural MRI is one of the most straight-forward neuroimaging methods and is clinically relevant, with increased ventricle volume and reductions in brain tissue, particularly grey matter, as known patterns of brain aging in humans [6–9]. MRI has shown that grey matter loss starts early and is gradual in humans [45, 46] and in rodents [31, 47–49]. As shown here, cortical thinning and increased ventricle volumes were found in middle-aged and old mice with T2W MRI (Figs. 1, 3).

### The relationship between CMBs, AD, and stroke

The neurovascular unit (NVU), the coupling between neural activity and blood flow, exemplifies the intimate relationship between the brain and its vessels and plays an important role in aging, AD progression, and stroke [50]. CMBs, biomarkers of cerebral small vessel disease, are hypothesized to represent NVU dysfunction mediated by leaks in the BBB, that lead to microvasculature rupture and bleeding [51]. In the Rotterdam study, a prospective study of a general population aged ≥ 45 years and currently the most comprehensive study for understanding the impact of CMBs on human disease, CMBs were found to have a prevalence of 15.3% and the increased presence of CMBs were associated with cognitive decline [52]. Another pooled study based on a random effects model found that the prevalence of CMBs in AD is 23% [53]. These studies [52, 53] have also demonstrated a higher prevalence of CMBs with stroke incidence, a leading cause of death in the US and in low-middle income countries [54]. The thalamus for example is a common deep location for CMBs presentation and is related to future risk of deep intracerebral hemorrhage [20, 28, 55]. The composite of these human studies has led to the conclusion that CMBs are significant radiologic findings, predictive of future stroke, cognitive decline, dementia, and possibly certain forms of AD, with pathological changes of cerebral small vessels [20, 52]. Yet, the mechanisms of CMBs formation is diverse and requires further investigation beyond association studies. For example, it is possible that when CMBs become very large and numerous or the BBB is not intact, then the risk of stroke is high. In addition, this damage could become cumulative and may promote future risk for cognitive decline in the form of dementia and AD.

CMBs have been detected by histology in non-TG mice [56, 57] and by MRI in amyloid precursor protein (APP) TG mouse models of AD [26, 58]. Although accurate in terms of specificity of stains and high image resolution, histology methods lead to selection bias due to the tedious nature of sample preparation and known artifacts of histology. High-field MRI is an alternative method that enables non-invasive and non-perturbing whole-brain imaging, mitigating selection bias, although it does require access to highly specialized equipment. Using high-field MRI, CMBs were observed in vivo with T2*W images, and were most frequently located in the thalamus (Fig. 2). This is in contrast to previous studies in APP TG mice, where CMBs were located in the neocortex and, to a lesser degree in the thalamus, [26] likely because of the different mechanisms of progression in cerebral amyloid angiopathy related CMBs [20, 27, 28]. Individual CMBs were visible in high resolution ex vivo T2*W MR images, enabling counting of individual CMBs sites in isotropic images (Fig. 5). Perfusion did not remove the CMBs pool during ex vivo imaging, indicating that the leaks are chronic iron deposits. CMBs in the cortex were also observed in the old-aged mice (Fig. 4). The cortical microhemorrhage site shown was larger than other CMBs commonly observed, and so was visible with DTI.

The thalamus is an important deep brain relay center that is highly vascularized (Fig. 6b, c). The thalamus includes major vascular connections via the internal carotid (IC), with the dorsal and ventral thalamic arteries, the posterior cerebral artery (PCA), with the thalamoperforating arteries, and the posterior communicating artery (PcomA), with the ventral thalamic arteries [39]. Moreover, in the study of whole brain vasculature by Xiong et al. in C57BL/6J mice, the capillary densities of the thalamus and cortex were shown to be relatively high, and it was found that the thalamus contains a relatively large proportion of large-sized (> 20 μm) vessel density related to other brain regions in the study [59]. The study by Xiong et al., corroborated the anatomical findings of Dorr et al. for the analysis of brain vasculature in mice and indicated that the combined presence of large vessels and small capillaries may make the thalamus highly vulnerable to CMBs with vascular aging.

### Diffusion imaging in the aging brain

DTI is best known for characterization of white matter damage after stroke or other white matter abnormalities, including ischemia, demyelination, axonal damage, inflammation, and edema [60, 61]. While advances better utilize large numbers of magnetic-field gradient directions, including diffusion spectrum imaging (DSI) or model-free Q-space methods [38, 62–66], DTI remains the most common method, with metrics comparable across studies. Focusing on clinical applications of DTI in neurology, Moseley et al. found significant declines in white matter organization occured in normal as well as in abnormal aging, indicating non-specific breakdown in the microstructure of the white matter, including demyelination, deterioration, and axonal loss [67]. A common concept in behavioral neurology is that DTI metric changes in the brain accelerate with disease, including measured FA, RD, and MD. In the article by Stebbins et al., it is stated that further increases in MD and RD with decreases in FA are common with progression of either AD or mild cognitive impairment [68]. More specifically, lower FA indicates loss of directional diffusion, possibly associated with axonal damage, while higher MD or RD indicate increased mean translational or radial diffusion, respectively, and are often associated with damage to myelin [68]. Other studies generally agree with these general trends in the DTI metric changes with aging, in humans [16, 69–71] and in a mouse model of AD [49].

In this current study, our ex vivo sequence was exactly the same for young and old mice. Our data show that the whole brain or sub-regions can be extracted in images from DTI (Fig. 4). The sites of particular interest were the thalamus and the cortex where aging biomarkers were observed, for example cortical thinning (Fig. 1) and CMBs (Fig. 2). Second, with statistical analysis in these regions, decreased FA and increased MD or RD (old vs. young) was apparent.

### Rationale for MRI in vivo and ex vivo

There are multiple reasons for performing combined in vivo and ex vivo MRI with advantages and disadvantages for both. These include, differences in tissue properties, the skull (in vivo) and cutting the brain out of the skull (ex vivo), the use of perfusion and the presence of PFA ex vivo, and the advantage of extended imaging time with higher resolution ex vivo. For example, ex vivo 3D T2*W imaging enabled a decrease in voxel volume of 16.5X (isotropic 140 µm^3^ versus 55 µm^3^), and high SNR enabled by a small RF coil that is close to the sample. Using the T2*W MRI method performed in vivo, it was difficult to count individual CMBs, because they appear as agglomerates (Fig. 2). Ex vivo imaging made it was possible to count individual CMBs because the signal and resolution was much higher (Fig. 5) and the size and shape of CMBs visualized in T2*W ex vivo MRI related more closely to histology (Fig. 6a). For ex vivo DTI, there may be concerns about PFA perfusion, and how that will impact the measurements being made. Yet, previous studies have shown that similar DTI measurements can be performed ex vivo by using freshly excised tissues, soaking tissues in buffer solution (to remove PFA), and by increasing the diffusion B-value to account for the reduced overall proton diffusion after fixation [72–74].

### Mechanisms of CMBs formation during aging associated with aortic stiffness

In both rodents and humans the stiffness of the proximal aorta increases with age, augmenting systolic blood pressure (BP) pulsatility and increasing the risk of CV events [21–23]. The proximal aorta is hypothesized to be a shock absorber, but with age, high pulses of pressure that can damage the brain microvasculature are sent to the small delicate blood vessels downstream [24, 25]. CMBs are visible in MRI and are thought to be a consequence of high pulses of systolic BP propagating into the small vessels of the brain. The high pulses of pressure from the stiffened aorta could damage the cerebral vessel walls in a way that increases leaking of blood through the wall in vulnerable brain regions, accelerating neurodegeneration.

## Conclusions

CMBs are brain biomarkers associated with poor clinical and cognitive outcomes in humans. Translational imaging approaches in normal mice simply age-matched to older human counterparts were used to identify CMBs, along with structural and diffusion changes in the brain. Our results stand to enhance our understanding of biological indicators in age-related diseases through in vivo*/*ex vivo correlation, provide a greater understanding of aging’s effect on neurovascular coupling, and could serve useful in the planning or monitoring of future pre-clinical studies with investigational interventions.

## Data Availability

Data will be made available on reasonable request from the corresponding authors.
